# The Dual Role of Inflammation in Colon Carcinogenesis

**DOI:** 10.3390/ijms130911071

**Published:** 2012-09-06

**Authors:** Giovanni Monteleone, Francesco Pallone, Carmine Stolfi

**Affiliations:** Department of Systems Medicine, University of Rome “Tor Vergata”, Via Montpellier 1, Rome 00133, Italy; E-Mails: gi.monteleone@med.uniroma2.it (G.M.); pallone@uniroma2.it (F.P.)

**Keywords:** colitis-associated colon cancer, IBD, ulcerative colitis, AOM + DSS, tumor immunosurveillance, T cells, inflammation, IL-21, IL-6, IL-17

## Abstract

Chronic inflammation characterizing patients with inflammatory bowel disease (IBD) represents a major risk factor for the development of colorectal cancer. Mechanisms underlying this neoplastic transformation are not fully understood though studies in experimental models of colon carcinogenesis suggest that inflammatory cell-derived cytokines either directly or indirectly stimulate the uncontrolled growth of cancer cells. Nevertheless, under specific inflammatory conditions, immune cells can boost an anti-tumor immune response with the down-stream effect of eliminating dysplastic and cancerous cells. This review outlines the beneficial and detrimental role of inflammation in colon carcinogenesis.

## 1. Introduction

Chronic inflammation is supposed to be a driving force for the growth of many human cancers [1]. This is for example evident in patients with ulcerative colitis (UC) and patients with Crohn’s disease (CD), the two major forms of inflammatory bowel disease (IBD) in humans. Indeed, the natural history of IBD patients, and particularly UC patients, can be marked by the development of colorectal cancer (CRC) [2,3]. There is also evidence that the risk of IBD-associated CRC is strictly linked to the duration and extension of inflammation [3,4], even though we do not yet know why CRC occurs only in a minority of IBD patients. Nonetheless, a considerable amount of work has been recently produced to show that mucosal inflammatory cell types can have effects either promoting (e.g., regulatory T cells (Tregs), Type 2 macrophages, CD4+ T helper (Th)-17 cells) or inhibiting (e.g., CD8+ T cells, Natural killer (NK) cells) CRC cell growth [5,6]. Moreover, inflammatory cells may modulate the process of colon carcinogenesis by stimulating stromal cells to synthesize a vast array of molecules with mitogenic effects on CRC cells [7]. The regulatory effects of inflammatory cells on the growth and survival of cancer cells are in part dependent on the synthesis of cytokines, which can either directly or indirectly target CRC cells and modulate their behavior. Cytokines such as interleukin (IL)-6, IL-17A, IL-21 and tumor necrosis factor (TNF)-α contribute to the formation of a tumor-supportive microenvironment, while interferon (IFN)-γ is supposed to exert tumor-suppressive functions [8]. It is thus conceivable that the type of inflammatory infiltrate can be a major determinant in the initiation and progression of colon carcinogenesis. In this article, we discuss the beneficial and detrimental role of gut inflammation in the growth of CRC cells.

## 2. Dual Role of Immune Cells in IBD-Associated CRC

Although the development of experimental models of IBD-associated CRC has advanced our knowledge of the pathogenesis of this neoplasia, the distinction between tumor-promoting and tumor-suppressive mucosal inflammation is still not completely clear. This is partly due to the fact that many immune cell subsets can be potentially bi-functional during the pathogenesis of this neoplasia and exert both tumor-promoting and tumor suppressive activities in response to the selective pressure of the surrounding microenvironment, a phenomenon known as immune-editing [9]. IBD-related CRC is characterized by a dense infiltrate of both innate immune cells, such as macrophages, neutrophils, myeloid derived suppressor cells (MDSC), dendritic cells (DC) and NK cells, and adaptive immune cells, such as T and B lymphocytes. The anti-tumor and tumor-promoting mechanisms of these cell subsets are summarized in Table 1 and discussed below.

### 2.1. CD4+ Th Cells

Tumor infiltrating CD4+ Th lymphocytes can support an effective host anti-tumor immune response against sporadic CRC [10]. Contrarily, these cells are critical for the maintenance of chronic inflammation in IBD patients and could promote IBD-associated CRC. The role of the Th1 and Th2 response in colitis-associated CRC was assessed by Osawa and coworkers [11]. By using an experimental model that mimics CD-associated CRC, in which mice received intrarectal administration of trinitrobenzene sulfonic acid (TNBS) and intraperitoneal injection of the carcinogen azoxymethane (AOM), these authors show that mice deficient in IFN-γ, develop significantly more neoplasms compared to wild-type mice and Th2-biased IL4−/− mice. Due to the high expression of Th2-derived cytokines (*i.e*., IL-4 and IL-5) observed in IFNγ−/− mice, the authors suggest that a Th2-dominant cytokine response may enhance CRC growth [11]. Indeed, the Th2 response correlates with sporadic CRC progression both in pre-clinical models and in humans [12,13]. Additionally, the Th2 response could directly participate in colitis-associated tumor initiation since Th2-related cytokines (*i.e*., IL-4 and IL-13) increase the expression of activation-induced cytidine deaminase (AID), an enzyme, which induces DNA mutation in cultured colonic epithelial cells [14]. Moreover, the increased susceptibility of IFNγ−/− mice to colitis-associated CRC could also rely on the reduced anti-tumor activity because IFN-γ activates cytotoxic NK and CD8+ T cells [15]. These observations in mice are consistent with the demonstration that the Th1 response associates with an improved prognosis in many cancers [16]. However, an exaggerated Th1 immune response does not necessarily associate with a reduced risk of CRC. This is for example seen in patients with colonic CD, who have an enhanced risk of CRC despite high levels of Th1 cytokines in the gut [17]. Recent advances in the pathogenesis of CD and the discovery that the inflamed gut of CD patients contains high levels of Th17-type cytokines (e.g., IL-17A, IL-21, IL-22) [18], which are mitogenic for epithelial cancer cells, could explain this apparent contradiction.

### 2.2. Cytotoxic T Cells

CD8+ T cells and natural killer T (NKT) cells are immune cell subsets with cytotoxic activity on cancer cells. Presentation of tumor-specific antigens by antigen-presenting cells to CD8+ T cells results in release of different cytotoxic molecules (e.g., perforin, granzyme A, granzyme B, TNF-related apoptosis-inducing ligand (TRAIL), Fas ligand) which can target and kill dysplastic and tumor cells [19]. Activated CD8+ T cells also produce IFN-γ, known to play a crucial role in amplifying the anti-tumor immune response [20]. Although infiltration of CD8+ T cells has been associated with an improved prognosis in many cancers, including CRC [21,22], data on the host immune response against IBD-related CRC are limited and somehow controversial. By comparing samples taken from patients with colitis-associated and sporadic CRC, Michael-Robinson and coworkers showed that, despite the fact that inflammation-associated lesions had an increased infiltration of CD8+ T cells, this was not associated with an improved prognosis [23]. This can be related to the fact that CD8+ T cells are implicated not only in cancer immunosurveillance but also in the pathogenesis of IBD. Indeed, increased infiltration with CD8+ T cells in the intestinal mucosa of active CD and UC correlates with elevated expression of perforin and granzyme which may sustain a tumor-promoting chronic inflammation [24]. In accordance with this hypothesis, by using a well-known experimental model of colitis-associated CRC, induced by AOM and dextran sodium sulfate (DSS), Waldner *et al*. reported that perforin-deficient mice developed a less severe colitis and fewer tumors than wild-type mice [25]. On the other hand, work from our laboratory showed that mice over-expressing Smad7 in T and NKT cells developed a severe colitis following AOM + DSS administration, which reduced the tumor burden. This protection was associated with increased expression of IFN-γ and increased accumulation of cytotoxic CD8+ T and NKT cells in the tumors and peritumoral areas [26]. A typical example of bi-functional immune cell subset in CRC growth is represented by NKT cells [27]. Besides cytotoxic activity, these cells can express Th1-, Th2- and Th17-related cytokines and regulate the immune response. Different and even the same NKT cell subsets can act as enhancers or suppressors of tumor immunity. Type I NKT cells, which express an invariant TCR-α chain (*i.e*., Vα24Jα18-Vβ11 in humans and Vα14Jα18-Vβ8 in mice), potentiate anti-tumor immunity by inducing IFN-γ expression [28], and tumor infiltration by Type I NKT cells in CRC patients has been positively correlated with disease-free survival [27]. Contrary, Type II NKT cells, which do not express this invariant TCR-α chain, associate with suppression of tumor immune surveillance in different cancer models [27]. Regarding IBD, activation of Type II NKT cells and the following production of the immunosuppressive cytokine IL-13 is a feature of the “atypical” Th2 response characterizing UC patients [29]. Additionally, an aberrant Type II NKT cell response was recently reported to directly contribute to intestinal inflammation in mice [30]. Although there is no conclusive evidence that Type II NKT cells contribute to colitis-associated CRC, it is tempting to speculate that activation of this cell subset in course of colitis may contribute to colon carcinogenesis by both dampening the host anti-tumor response and amplifying the ongoing inflammation.

### 2.3. Regulatory T Cells

CD4+ T cells expressing CD25 and the master transcription factor Foxp3 (CD4+CD25+Foxp3+), termed regulatory T cells (Tregs), exert immunosuppressive effects both via direct cell–cell interactions and through the release of the cytokines IL-10 and TGF-β [31]. Whereas Tregs are important in limiting autoimmune diseases and inflammatory responses, the suppression of the immune system may strongly hamper host immune surveillance against tumors. The pro-tumoral role of Tregs in the progression of established tumors is well accepted and Foxp3 expression has been associated with a poor prognosis in many cancer types [32]. Contrarily, in patients bearing CRC, infiltration of Tregs associates with a favorable prognosis [33]. Consistently, a study by Erdman and coworkers showed that Tregs reduce tumor growth in ApcMin/+ mice, which spontaneously develop intestinal lesions [34]. The role of Tregs in inflammation-associated CRC remains elusive. Given the potent immunoregulatory function Tregs exert on immune responses and inflammation, it is conceivable that these cells may help prevent and/or delay inflammation-mediated tumor growth. In line with this hypothesis, a recent work by Sugai and colleagues suggested that Tregs exert anti-tumor activity in colitis-associated CRC [35]. These authors showed that mice deficient for Runx3, a protein required for proper differentiation and function of Tregs, are more susceptible to develop colitis and inflammation-related colonic tumors compared to wild-type mice [35]. However, further investigation is needed to address the role of Tregs in IBD-related CRC.

### 2.4. Innate Immune Cells

Cells of the innate immune system, such as neutrophils, NK cells, DC and macrophages infiltrate colitis-associated CRC. Whereas the contribution of innate immunity in CRC initiation through oxidative stress is well accepted [1], the role of these cells in colitis-related CRC growth has recently started to be unveiled. A link between innate immunity and this neoplasia is suggested by the demonstration that Toll-like receptors (TLRs) are involved in inflammation-related carcinogenesis. TLRs are a family of membrane-bound receptors mainly expressed by cells of the innate immune system and able to recognize microbe specific molecules or endogenous stress signals. TLRs play a major role in maintaining gut homeostasis, and polymorphisms in the genes encoding TLRs are associated with an increased risk of IBD [36]. Additionally, TLR4 is up-regulated in intestinal epithelial cells of patients with active IBD [37] and TRL4-driven signals induce ROS production and mitogenic molecules (e.g., Prostaglandin E2) [38]. Experimental models of colitis-associated CRC showed that the presence and recognition of the gut microbiota are required for inflammation-associated carcinogenesis [38]. Fukata and coworkers report that TLR4 is over-expressed in UC-associated CRC and, by using the AOM + DSS mouse model, these authors showed that TLR4-deficient mice are largely protected against the development of tumors as compared to wild-type mice [39]. More recently, the same authors suggested that innate immune signaling by TLR4 could shape the inflammatory microenvironment to sustain CRC cell growth [40]. Contrarily, myeloid differentiation factor 88 (MyD88), a molecule critical for TLR intracellular signaling, seems to have a protective role against inflammation-related CRC induced in mice by AOM + DSS treatment [41]. However, it is worth noting that MyD88 signaling has been implicated in cancer promotion in different mouse models of CRC [42,43]. Important players in colitis-associated CRC growth are tumor associated macrophages (TAM), which can be divided into M1 and M2 types [44]. M1 macrophages, activated by IFN-γ and microbial products, are capable of killing pathogens and priming antitumor immune responses through the up-regulation of major histocompatibility complex (MHC) molecules and the production of pro-inflammatory cytokines (e.g., TNF-α, IL-6, IL-12). Contrary, M2 or “alternatively activated macrophages”, which are induced *in vitro* by IL-4, IL-10, and IL-13, are known to support angiogenesis and inhibit anti-cancer immunity through the production of TGF-β and IL-10. However, although most TAM are considered to have an M2 phenotype and promote inflammation-related CRC, most tumor-promoting cytokines are “M1 cytokines” [45]. In addition to TAM, MDSC are suggested to promote colitis-associated CRC by dampening anti-tumor immune responses [45]. A possible role for innate immune cells in the pathogenesis of this cancer type was highlighted by Hayakawa and coworkers, who showed that ASK1(−/−) mice, in which macrophages are impaired in their ability to kill bacteria, develop a more severe colitis and more numerous and larger tumors than wild-type mice following AOM + DSS treatment [46].

## 3. Role of Cytokines in IBD-Associated CRC

The cross-talk between immune cells and CRC cells is mostly mediated by cytokines, which may exert both pro-tumor and anti-tumor effects [47].

### 3.1. TNF-α

TNF-α, mainly produced by activated M1 macrophages and T lymphocytes, plays a pivotal role in the inflammatory process by binding TNF receptors (TNF-R) p55 and p75 on target cells. Reports on the role of TNF-α in cancer suggest that the cytokine may either promote or inhibit CRC cell growth [48]. Inflammatory cells may use a TNF-α outburst to stimulate ROS and kill transformed cells. On the other hand, chronic production of low doses TNF-α could sustain tumor growth [48]. TNF-α is crucial in the pathogenesis of colitis and the use of anti-TNF-α monoclonal antibodies is beneficial in IBD patients [49]. Similarly, TNF-α signaling seems to be involved in colitis-associated CRC. Popivanova and coworkers showed that TNF-Rp55−/− mice treated with AOM + DSS developed a milder colitis and fewer tumors than wild-type mice [50]. It was also shown that wild-type mice transplanted with bone marrow of TNF-Rp55-deficient mice developed significantly fewer tumors after AOM + DSS treatment than either wild-type mice or TNF-Rp55−/− mice transplanted with wild-type bone marrow. Moreover, mice given anti-TNF-α were less susceptible to both colitis and colitis-associated CRC [50]. As TNF-α is a powerful inducer of NF-kB, a pleiotropic transcription factor supporting both inflammation and carcinogenesis [51] and blockade of NF-kB activation in the intestinal epithelium significantly reduces the incidence of colitis-associated tumors [52], the pro-inflammatory and tumor-promoting effects of TNF-α in this model could be mediated by NF-kB.

### 3.2. IL-6

IL-6 is a multifunctional cytokine produced by T cells, B cells, macrophages and fibroblasts with a crucial role in the generation of Th17 cells [53]. IL-6 also regulates epithelial cell proliferation and survival either by binding to the membrane-bound IL-6 receptor (IL-6Rα/gp130) on target cells or to soluble IL-6Rα (sIL-6Rα) with subsequent trans-signaling [54]. These receptors initiate a signal cascade leading to activation of signal transducer and activator of transcription (STAT)3, with the downstream effect of inducing anti-apoptotic and proliferative genes [55,56]. However, it is worth noting that two recent studies, performed in ApcMin/+ mice, showed STAT3 to exert opposite roles on colon carcinogenesis depending on the tumor stage [57,58]. IL-6 has been linked to IBD pathogenesis [59,60] and been suggested to play a pivotal role in colitis-associated CRC. Accordingly, in the AOM + DSS mouse model, Becker and colleagues demonstrate that IL-6 is highly produced by CD4+ T cells and promotes tumor cell proliferation via STAT3 activation [61]. In a similar model, Grivennikov and coworkers show that IL-6 expressed by lamina propria myeloid cells protects normal and malignant epithelial cells from apoptosis in a STAT3-dependent fashion [56]. An essential role of IL-6 trans-signaling in the development of colitis-associated CRC is also reported by Matsumoto and colleagues [62]. Recently, Li *et al*. found higher expressions of IL-6 and STAT3 in both patients with active UC and those who had progressed to CRC, compared with patients with inactive disease or control patients [63]. In the same study, the expression of the suppressor of cytokine signaling (SOCS)3, a protein that hampers the transcription of IL-6-target genes, is found to be decreased in UC patients who had developed CRC, thus suggesting an important role for SOCS3 in UC-related CRC [63]. Consistently, in the AOM + DSS model, mice with deletion of SOCS3 in intestinal epithelial cells show increased STAT3 and NF-κB activation and enhanced susceptibility to developing colonic tumors following AOM + DSS treatment [64]. Finally, Gerlach and colleagues demonstrate a critical role for the transcription factor NFATc2 in IL-6 signaling and colitis-associated CRC [65]. In this study, NFATc2-knockout mice produced reduced IL-6 and were almost completely protected from the AOM + DSS-induced tumors, in contrast to wild-type mice, which bore multiple colonic lesions. Notably, administration of hyper-IL-6 abrogated protection from tumor progression in NFATc2-deficient mice and restored tumor incidence to levels observed in wild-type mice [65].

### 3.3. IL-17A

IL-17A, mainly secreted by Th17 cells and, to a lesser extent, by NKT cells, innate lymphoid cells and γδ T cells, exerts pro-inflammatory properties essential for the host protection against extracellular pathogens [66]. On the other hand, IL-17A has been implicated in the pathogenesis of many chronic inflammatory disorders and clinical trials with IL-17A-neutralizing antibodies have reported benefit in patients with psoriasis and rheumatoid arthritis [67]. However, IL-17A blockade was not beneficial in CD patient populations [68]. Consistent with this observation is also the demonstration that transfer of IL-17A-deficient T cells to immunocompromized mice promotes a colitis which is indistinguishable from that induced by wild-type T cells [69]. Similarly, studies in cancer models have shown a dual role of IL-17A in controlling neoplastic cell growth. Indeed, IL-17A inhibits tumor growth in a murine model of melanoma [70] and in implanted tumor models [71,72], while promotes malignant cell growth in mouse models of spontaneous intestinal cancer [73,74]. Using the AOM + DSS model, Hyun and colleagues showed that IL-17A-deficient mice developed a less severe colitis than wild-type mice, as demonstrated by the decreased cell infiltrate and the diminished expression of pro-inflammatory factors (e.g., TNF-α, IL-6) [75]. Consistently, in IL-17A-knockout mice STAT3 activation was markedly reduced compared with wild-type mice, and this associated with the development of fewer and smaller colonic tumors [75].

### 3.4. IL-21

IL-21 is a pleiotropic cytokine produced by activated Th and NKT cells and T follicular helper cells [76]. Both pre-clinical and clinical studies have shown that IL-21 has potent anti-tumor effects due to its ability to expand the cytotoxic immune response [77]. However, IL-21 has been involved in the pathogenesis of many immune-mediated diseases including IBD [76]. Indeed, IL-21 is over-produced in the colonic mucosa of UC and CD patients [78] where it positively regulates Th17 cell responses [79]. Notably, two independent and recent studies revealed a key role for this cytokine in promoting colitis-associated CRC [80,81]. High levels of IL-21 have been observed in the gut of patients with UC-associated colon cancer and in mice with colitis-associated CRC induced by AOM + DSS [80]. In this model, IL-21-deficient mice developed a less severe colitis than wild-type mice, characterized by reduced mucosal damage, reduced infiltration of T cells, and diminished production of IL-6 and IL-17A. IL-21KO mice also developed fewer and smaller tumors compared with control mice [80]. Analysis of mechanisms underlying this effect revealed that IL-21 sustains CD4+ T cell infiltration in the tumor and peritumor areas and enhances the production of IL-6 and IL-17A as well as STAT3 activation. Moreover, colonic tumors developing in wild-type mice exhibited an increased infiltrate of both M2 macrophages and MDSC than those grown in IL-21-deficient mice [80]. In a very similar model, Fichtner-Feigl’s group showed that IL-21 promotes colitis-related CRC and associated this effect with increased tumor cell proliferation and impaired anti-tumor response of CD103+ CD8+ cytotoxic T cells specific for cancer cells [81].

## 4. Conclusions

Clinical and experimental data indicate that chronic colitis increases the risk of developing tumors. However, it is now clear that tumor-promoting inflammation and anti-tumor immunity coexist in colitis-associated CRC. Rising evidence suggests that the specific nature of tumor infiltrating inflammatory cells and their derived cytokines may determine the beneficial versus the detrimental effects of inflammation in the pathogenesis of this neoplasia. Understanding the mechanisms by which the immune system can tip this balance towards one side or the other could help reduce potential drawbacks in future anti-tumor immune therapy and develop novel approaches for treating IBD-related CRC.

## Figures and Tables

**Table 1:** Dual role of immune cells in inflammatory bowel disease (IBD)-related colorectal cancer (CRC).

Immune cell types	Tumor-promoting	Tumor-suppressive
Macrophages, dendritic cells, myeloid-derived suppressor cells	Immunosuppression, production of cytokines (e.g., TNF-α, IL-6)	Antigen presentation, production of cytokines (e.g., IL-12, IFN-γ, TNF-α)
Neutrophils	Oxidative stress, production of cytokines	Direct cytotoxicity towards cancer cells, production of ROS
Natural killer (NK) cells	-	Direct cytotoxicity towards cancer cells, production of IFN-γ and cytotoxic molecules
NKT cells	Immunosuppression, production of cytokines	Direct cytotoxicity towards cancer cells, production of IFN-γ and cytotoxic molecules
CD8+ T cells	Production of cytokines, production of perforin	Direct cytotoxicity towards cancer cells, production of IFN-γ and cytotoxic molecules
CD4+ Th1 cells	-	Production of IFN-γ, help to CD8+ T cells in tumor rejection
CD4+ Th2 cells	Production of cytokines (e.g., IL-13)	-
CD4+ Th17 cells	Production of cytokines (e.g., IL-17, IL-21, IL-23, IL-6)	Activation of cytotoxic T lymphocytes, production of cytokines
CD4+ Tregs	Immunosuppression	Suppression of inflammation

## References

[1] Kundu J.K., Surh Y.J. (2008). Inflammation: Gearing the journey to cancer. Mutat. Res.

[2] Bernstein C.N., Blanchard J.F., Kliewer E., Wajda A. (2001). Cancer risk in patients with inflammatory bowel disease: A population-based study. Cancer.

[3] Eaden J.A., Abrams K.R., Mayberry J.F. (2001). The risk of colorectal cancer in ulcerative colitis: A meta-analysis. Gut.

[4] Ekbom A., Helmick C., Zack M., Adami H.O. (1990). Ulcerative colitis and colorectal cancer. A population-based study. N. Engl. J. Med.

[5] Rizzo A., Pallone F., Monteleone G., Fantini M.C. (2011). Intestinal inflammation and colorectal cancer: A double-edged sword?. World J. Gastroenterol.

[6] Erreni M., Mantovani A., Allavena P. (2011). Tumor-associated macrophages (tam) and inflammation in colorectal cancer. Cancer Microenviron.

[7] Mueller M.M., Fusenig N.E. (2004). Friends or foes—Bipolar effects of the tumour stroma in cancer. Nat. Rev. Cancer.

[8] Terzic J., Grivennikov S., Karin E., Karin M. (2010). Inflammation and colon cancer. Gastroenterology.

[9] Grivennikov S.I., Greten F.R., Karin M. (2010). Immunity, inflammation, and cancer. Cell.

[10] Galon J., Costes A., Sanchez-Cabo F., Kirilovsky A., Mlecnik B., Lagorce-Pages C., Tosolini M., Camus M., Berger A., Wind P. (2006). Type, density, and location of immune cells within human colorectal tumors predict clinical outcome. Science.

[11] Osawa E., Nakajima A., Fujisawa T., Kawamura Y.I., Toyama-Sorimachi N., Nakagama H., Dohi T. (2006). Predominant t helper type 2-inflammatory responses promote murine colon cancers. Int. J. Cancer.

[12] Kettunen H.L., Kettunen A.S., Rautonen N.E. (2003). Intestinal immune responses in wild-type and apcmin/+ mouse, a model for colon cancer. Cancer Res.

[13] Shibata M., Nezu T., Kanou H., Abe H., Takekawa M., Fukuzawa M. (2002). Decreased production of interleukin-12 and type 2 immune responses are marked in cachectic patients with colorectal and gastric cancer. J. Clin. Gastroenterol.

[14] Endo Y., Marusawa H., Kou T., Nakase H., Fujii S., Fujimori T., Kinoshita K., Honjo T., Chiba T. (2008). Activation-induced cytidine deaminase links between inflammation and the development of colitis-associated colorectal cancers. Gastroenterology.

[15] Dunn G.P., Koebel C.M., Schreiber R.D. (2006). Interferons, immunity and cancer immunoediting. Nat. Rev.

[16] Swann J.B., Smyth M.J. (2007). Immune surveillance of tumors. J. Clin. Investig.

[17] Canavan C., Abrams K.R., Mayberry J. (2006). Meta-analysis: Colorectal and small bowel cancer risk in patients with crohn’s disease. Aliment. Pharmacol. Ther.

[18] Monteleone I., Sarra M., Pallone F., Monteleone G. (2012). Th17-related cytokines in inflammatory bowel diseases: Friends or foes?. Curr. Mol. Med.

[19] Dunn G.P., Old L.J., Schreiber R.D. (2004). The immunobiology of cancer immunosurveillance and immunoediting. Immunity.

[20] Tannenbaum C.S., Hamilton T.A. (2000). Immune-inflammatory mechanisms in ifngamma-mediated anti-tumor activity. Semin. Cancer Biol.

[21] Talmadge J.E., Donkor M., Scholar E. (2007). Inflammatory cell infiltration of tumors: Jekyll or hyde. Cancer Metastasis Rev.

[22] Naito Y., Saito K., Shiiba K., Ohuchi A., Saigenji K., Nagura H., Ohtani H. (1998). CD8+ T cells infiltrated within cancer cell nests as a prognostic factor in human colorectal cancer. Cancer Res.

[23] Michael-Robinson J.M., Pandeya N., Walsh M.D., Biemer-Huttmann A.E., Eri R.D., Buttenshaw R.L., Lincoln D., Clouston A.D., Jass J.R., Radford-Smith G.L. (2006). Characterization of tumour-infiltrating lymphocytes and apoptosis in colitis-associated neoplasia: Comparison with sporadic colorectal cancer. J. Pathol.

[24] Muller S., Lory J., Corazza N., Griffiths G.M., Z’Graggen K., Mazzucchelli L., Kappeler A., Mueller C. (1998). Activated CD4+ and CD8+ cytotoxic cells are present in increased numbers in the intestinal mucosa from patients with active inflammatory bowel disease. Am. J. Pathol.

[25] Waldner M.J., Wirtz S., Becker C., Seidel D., Tubbe I., Cappel K., Hahnel P.S., Galle P.R., Schuler M., Neurath M.F. (2010). Perforin deficiency attenuates inflammation and tumor growth in colitis-associated cancer. Inflamm. Bowel Dis.

[26] Rizzo A., Waldner M.J., Stolfi C., Sarra M., Fina D., Becker C., Neurath M.F., Macdonald T.T., Pallone F., Monteleone G. (2011). Smad7 expression in T cells prevents colitis-associated cancer. Cancer Res.

[27] Berzofsky J.A., Terabe M. (2009). The contrasting roles of NKT cells in tumor immunity. Curr. Mol. Med.

[28] Kitamura H., Iwakabe K., Yahata T., Nishimura S., Ohta A., Ohmi Y., Sato M., Takeda K., Okumura K., van Kaer L. (1999). The natural killer T (NKT) cell ligand alpha-galactosylceramide demonstrates its immunopotentiating effect by inducing interleukin (IL)-12 production by dendritic cells and IL-12 receptor expression on NKT cells. J. Exp. Med.

[29] Fuss I.J., Heller F., Boirivant M., Leon F., Yoshida M., Fichtner-Feigl S., Yang Z., Exley M., Kitani A., Blumberg R.S. (2004). Nonclassical CD1d-restricted NK T cells that produce IL-13 characterize an atypical Th2 response in ulcerative colitis. J. Clin. Investig.

[30] Liao C.M., Zimmer M.I., Shanmuganad S., Yu H.T., Cardell S.L., Wang C.R. (2012). Dysregulation of cd1d-restricted type ii natural killer t cells leads to spontaneous development of colitis in mice. Gastroenterology.

[31] Thompson C., Powrie F. (2004). Regulatory T cells. Curr. Opin. Pharmacol.

[32] Deleeuw R.J., Kost S.E., Kakal J.A., Nelson B.H. (2012). The prognostic value of Foxp3+ tumor-infiltrating lymphocytes in cancer: A critical review of the literature. Clin. Cancer Res.

[33] Ladoire S., Martin F., Ghiringhelli F. (2011). Prognostic role of Foxp3+ regulatory T cells infiltrating human carcinomas: The paradox of colorectal cancer. Cancer Immunol. Immunother.

[34] Erdman S.E., Sohn J.J., Rao V.P., Nambiar P.R., Ge Z., Fox J.G., Schauer D.B. (2005). CD4+ CD25+ regulatory lymphocytes induce regression of intestinal tumors in Apcmin/+ mice. Cancer Res.

[35] Sugai M., Aoki K., Osato M., Nambu Y., Ito K., Taketo M.M., Shimizu A. (2011). Runx3 is required for full activation of regulatory T cells to prevent colitis-associated tumor formation. J. Immunol.

[36] Fukata M., Abreu M.T. (2008). What are Toll-like receptors and what role may they have in IBD?. Inflamm. Bowel Dis.

[37] Cario E., Podolsky D.K. (2000). Differential alteration in intestinal epithelial cell expression of Toll-like receptor 3 (TLR3) and TLR4 in inflammatory bowel disease. Infect. Immun.

[38] Fukata M., Abreu M.T. (2008). Role of Toll-like receptors in gastrointestinal malignancies. Oncogene.

[39] Fukata M., Chen A., Vamadevan A.S., Cohen J., Breglio K., Krishnareddy S., Hsu D., Xu R., Harpaz N., Dannenberg A.J. (2007). Toll-like receptor-4 promotes the development of colitis-associated colorectal tumors. Gastroenterology.

[40] Fukata M., Hernandez Y., Conduah D., Cohen J., Chen A., Breglio K., Goo T., Hsu D., Xu R., Abreu M.T. (2009). Innate immune signaling by toll-like receptor-4 (TLR4) shapes the inflammatory microenvironment in colitis-associated tumors. Inflamm. Bowel Dis.

[41] Salcedo R., Worschech A., Cardone M., Jones Y., Gyulai Z., Dai R.M., Wang E., Ma W., Haines D., O’HUigin C. (2010). Myd88-mediated signaling prevents development ofadenocarcinomas of the colon: Role of interleukin 18. J. Exp. Med.

[42] Rakoff-Nahoum S., Medzhitov R. (2007). Regulation of spontaneous intestinal tumorigenesis through the adaptor protein Myd88. Science.

[43] Uronis J.M., Muhlbauer M., Herfarth H.H., Rubinas T.C., Jones G.S., Jobin C. (2009). Modulation of the intestinal microbiota alters colitis-associated colorectal cancer susceptibility. PLoS One.

[44] Sica A. (2010). Role of tumour-associated macrophages in cancer-related inflammation. Exp. Oncol.

[45] Allavena P., Sica A., Garlanda C., Mantovani A. (2008). The Yin-Yang of tumor-associated macrophages in neoplastic progression and immune surveillance. Immunol. Rev.

[46] Hayakawa Y., Hirata Y., Nakagawa H., Sakamoto K., Hikiba Y., Otsuka M., Ijichi H., Ikenoue T., Tateishi K., Akanuma M. (2010). Apoptosis signal-regulating kinase 1 regulates colitis and colitis-associated tumorigenesis by the innate immune responses. Gastroenterology.

[47] Mantovani A., Allavena P., Sica A., Balkwill F. (2008). Cancer-related inflammation. Nature.

[48] Bertazza L., Mocellin S. (2010). The dual role of tumor necrosis factor (TNF) in cancer biology. Curr. Med. Chem.

[49] Rutgeerts P., van Assche G., Vermeire S. (2004). Optimizing anti-TNF treatment in inflammatory bowel disease. Gastroenterology.

[50] Popivanova B.K., Kitamura K., Wu Y., Kondo T., Kagaya T., Kaneko S., Oshima M., Fujii C., Mukaida N. (2008). Blocking TNF-alpha in mice reduces colorectal carcinogenesis associated with chronic colitis. J. Clin. Investig.

[51] Vallabhapurapu S., Karin M. (2009). Regulation and function of NF-kappab transcription factors in the immune system. Annu. Rev. Immunol.

[52] Greten F.R., Eckmann L., Greten T.F., Park J.M., Li Z.W., Egan L.J., Kagnoff M.F., Karin M. (2004). Ikkbeta links inflammation and tumorigenesis in a mouse model of colitis-associated cancer. Cell.

[53] Mangan P.R., Harrington L.E., O’Quinn D.B., Helms W.S., Bullard D.C., Elson C.O., Hatton R.D., Wahl S.M., Schoeb T.R., Weaver C.T. (2006). Transforming growth factor-beta induces development of the T(H)17 lineage. Nature.

[54] Rose-John S., Scheller J., Elson G., Jones S.A. (2006). Interleukin-6 biology is coordinated by membrane-bound and soluble receptors: Role in inflammation and cancer. J. Leukoc. Biol.

[55] Bollrath J., Phesse T.J., von Burstin V.A., Putoczki T., Bennecke M., Bateman T., Nebelsiek T., Lundgren-May T., Canli O., Schwitalla S. (2009). Gp130-mediated Stat3 activation in enterocytes regulates cell survival and cell-cycle progression during colitis-associated tumorigenesis. Cancer Cell.

[56] Grivennikov S., Karin E., Terzic J., Mucida D., Yu G.Y., Vallabhapurapu S., Scheller J., Rose-John S., Cheroutre H., Eckmann L. (2009). Il-6 and Stat3 are required for survival of intestinal epithelial cells and development of colitis-associated cancer. Cancer Cell.

[57] Musteanu M., Blaas L., Mair M., Schlederer M., Bilban M., Tauber S., Esterbauer H., Mueller M., Casanova E., Kenner L. (2010). Stat3 is a negative regulator of intestinal tumor progression in Apc(min) mice. Gastroenterology.

[58] Lee J., Kim J.C., Lee S.E., Quinley C., Kim H., Herdman S., Corr M., Raz E. (2012). Signal transducer and activator of transcription 3 (Stat3) protein suppresses adenoma-to-carcinoma transition in Apcmin/+ mice via regulation of Snail-1 (Snai) protein stability. J. Biol. Chem.

[59] Hyams J.S., Fitzgerald J.E., Treem W.R., Wyzga N., Kreutzer D.L. (1993). Relationship of functional and antigenic interleukin 6 to disease activity in inflammatory bowel disease. Gastroenterology.

[60] Atreya R., Mudter J., Finotto S., Mullberg J., Jostock T., Wirtz S., Schutz M., Bartsch B., Holtmann M., Becker C. (2000). Blockade of interleukin 6 trans signaling suppresses T-cell resistance against apoptosis in chronic intestinal inflammation: Evidence in crohn disease and experimental colitis *in vivo*. Nat. Med.

[61] Becker C., Fantini M.C., Schramm C., Lehr H.A., Wirtz S., Nikolaev A., Burg J., Strand S., Kiesslich R., Huber S. (2004). Tgf-beta suppresses tumor progression in colon cancer by inhibition of IL-6 *trans*-signaling. Immunity.

[62] Matsumoto S., Hara T., Mitsuyama K., Yamamoto M., Tsuruta O., Sata M., Scheller J., Rose-John S., Kado S., Takada T. (2010). Essential roles of IL-6 *trans*-signaling in colonic epithelial cells, induced by the IL-6/soluble-IL-6 receptor derived from lamina propria macrophages, on the development of colitis-associated premalignant cancer in a murine model. J. Immunol.

[63] Li Y., de Haar C., Chen M., Deuring J., Gerrits M.M., Smits R., Xia B., Kuipers E.J., van der Woude C.J. (2010). Disease-related expression of the IL6/Stat3/Socs3 signalling pathway in ulcerative colitis and ulcerative colitis-related carcinogenesis. Gut.

[64] Rigby R.J., Simmons J.G., Greenhalgh C.J., Alexander W.S., Lund P.K. (2007). Suppressor of cytokine signaling 3 (Socs3) limits damage-induced crypt hyper-proliferation and inflammation-associated tumorigenesis in the colon. Oncogene.

[65] Gerlach K., Daniel C., Lehr H.A., Nikolaev A., Gerlach T., Atreya R., Rose-John S., Neurath M.F., Weigmann B. (2012). Transcription factor NFATc2 controls the emergence of colon cancer associated with IL-6-dependent colitis. Cancer Res.

[66] Curtis M.M., Way S.S. (2009). Interleukin-17 in host defence against bacterial, mycobacterial and fungal pathogens. Immunology.

[67] Zhu S., Qian Y. (2012). IL-17/IL-17 receptor system in autoimmune disease: Mechanisms and therapeutic potential. Clin. Sci.

[68] Hueber W., Sands B.E., Lewitzky S., Vandemeulebroecke M., Reinisch W., Higgins P.D., Wehkamp J., Feagan B.G., Yao M.D., Karczewski M. (2012). Secukinumab, a human anti-IL-17a monoclonal antibody, for moderate to severe crohn’s disease: Unexpected results of a randomised, double-blind placebo-controlled trial. Gut.

[69] Leppkes M., Becker C., Ivanov I.I., Hirth S., Wirtz S., Neufert C., Pouly S., Murphy A.J., Valenzuela D.M., Yancopoulos G.D. (2009). Rorgamma-expressing Th17 cells induce murine chronic intestinal inflammation via redundant effects of IL-17a and IL-17f. Gastroenterology.

[70] Muranski P., Boni A., Antony P.A., Cassard L., Irvine K.R., Kaiser A., Paulos C.M., Palmer D.C., Touloukian C.E., Ptak K. (2008). Tumor-specific Th17-polarized cells eradicate large established melanoma. Blood.

[71] Benchetrit F., Ciree A., Vives V., Warnier G., Gey A., Sautes-Fridman C., Fossiez F., Haicheur N., Fridman W.H., Tartour E. (2002). Interleukin-17 inhibits tumor cell growth by means of a T-cell-dependent mechanism. Blood.

[72] Kryczek I., Wei S., Szeliga W., Vatan L., Zou W. (2009). Endogenous IL-17 contributes to reduced tumor growth and metastasis. Blood.

[73] Wu S., Rhee K.J., Albesiano E., Rabizadeh S., Wu X., Yen H.R., Huso D.L., Brancati F.L., Wick E., McAllister F. (2009). A human colonic commensal promotes colon tumorigenesis via activation of t helper type 17 T cell responses. Nat. Med.

[74] Chae W.J., Gibson T.F., Zelterman D., Hao L., Henegariu O., Bothwell A.L. (2010). Ablation of IL-17a abrogates progression of spontaneous intestinal tumorigenesis. Proc. Natl. Acad. Sci. USA.

[75] Hyun Y.S., Han D.S., Lee A.R., Eun C.S., Youn J., Kim H.Y. (2012). Role of IL-17a in the development of colitis-associated cancer. Carcinogenesis.

[76] Monteleone G., Pallone F., Macdonald T.T. (2009). Interleukin-21 as a new therapeutic target for immune-mediated diseases. Trends Pharmacol. Sci.

[77] Sondergaard H., Skak K. (2009). IL-21: Roles in immunopathology and cancer therapy. Tissue antigens.

[78] Monteleone G., Monteleone I., Fina D., Vavassori P., del Vecchio Blanco G., Caruso R., Tersigni R., Alessandroni L., Biancone L., Naccari G.C. (2005). Interleukin-21 enhances T-helper cell type I signaling and interferon-gamma production in crohn’s disease. Gastroenterology.

[79] Fina D., Sarra M., Fantini M.C., Rizzo A., Caruso R., Caprioli F., Stolfi C., Cardolini I., Dottori M., Boirivant M. (2008). Regulation of gut inflammation and Th17 cell response by interleukin-21. Gastroenterology.

[80] Stolfi C., Rizzo A., Franze E., Rotondi A., Fantini M.C., Sarra M., Caruso R., Monteleone I., Sileri P., Franceschilli L. (2011). Involvement of interleukin-21 in the regulation of colitis-associated colon cancer. J. Exp. Med.

[81] Jauch D., Martin M., Schiechl G., Kesselring R., Schlitt H.J., Geissler E.K., Fichtner-Feigl S. (2011). Interleukin 21 controls tumour growth and tumour immunosurveillance in colitis-associated tumorigenesis in mice. Gut.

